# Real-world evidence on the association of novel antidiabetic medication use with cancer risk and protective effects: a systematic review and network meta-analysis

**DOI:** 10.1177/20420986251335214

**Published:** 2025-04-21

**Authors:** Ahmed S. Kenawy, Yi-Shao Liu, Ayobami Aiyeolemi, Godwin Okoye, Chanhyun Park

**Affiliations:** Health Outcomes Division, College of Pharmacy, The University of Texas at Austin, Austin, TX, USA; Health Outcomes Division, College of Pharmacy, The University of Texas at Austin, Austin, TX, USA; Health Outcomes Division, College of Pharmacy, The University of Texas at Austin, Austin, TX, USA; Health Outcomes Division, College of Pharmacy, The University of Texas at Austin, Austin, TX, USA; Health Outcomes Division, College of Pharmacy, The University of Texas at Austin, 2409 University Avenue MC A1930, Austin, TX 78712, USA

**Keywords:** cancer, diabetes, DPP-4 inhibitors, GLP-1 agonists, network meta-analysis, SGLT2 inhibitors

## Abstract

**Background:**

Novel antidiabetic medications (SGLT-2 inhibitors, DPP-4 inhibitors, and GLP-1 agonists) are commonly used worldwide; however, the available research lacks definitive conclusions on their protective effects or potential risks on cancer.

**Objectives:**

Compared to other antidiabetics, our systematic review and network meta-analysis (NMA) aims to use real-world studies to assess the potential cancer risks or protective effects of these novel antidiabetics.

**Methods:**

We comprehensively searched PubMed, CINAHL, and Web of Science from their inception until November 30, 2023. We included observational studies examining at least one novel antidiabetics in the systematic review. The novel antidiabetics include sodium-glucose cotransporter-2 inhibitors (SGLT-2i), dipeptidyl peptidase-4 inhibitors (DPP-4i), and glucagon-like peptide-1 agonists (GLP-1a).

**Design:**

We focused on cohort studies that provided data on cancer incidence and sample size in the NMA. Using NetMetaXL^®^, the random effects model with informative priors was used in the NMA to estimate the pooled odds ratio (OR) with 95% credible intervals (CrI).

**Results:**

The systematic review included 62 studies, of which 22 met the inclusion criteria for the NMA. SGLT-2i users had lower overall cancer risk compared to sulfonylureas (OR: 0.54; 95% CrI: 0.40–0.74, low certainty), GLP-1a (OR: 0.70; 95% CrI: 0.53–0.92, low certainty), and DPP-4i users (OR: 0.72; 95% CrI: 0.57–0.92, very low certainty). DPP-4i users also had a lower cancer risk than sulfonylureas users (OR: 0.76; 95% CrI: 0.60–0.96, low certainty). No other statistically significant ORs were found in other direct comparisons.

**Conclusion:**

SGLT-2i users have a lower risk of developing cancers than sulfonylureas, GLP-1a, and DPP-4i users. These results may improve patient safety by guiding future clinical practice and medication choices. Future studies should investigate the mechanisms behind these observed associations.

**Trial registration:**

This NMA was registered in PROSPERO (CRD42023469941).

## Plain language summary

Real-world evidence on the association of novel antidiabetic medication use with cancer risk and protective effects

Why we conducted this study?

Insufficient evidence exists regarding the potential effect of the new types of diabetes medications, known as SGLT-2 inhibitors, DPP-4 inhibitors, and GLP-1 agonists, on the risk of cancer. This study aimed to determine whether the use of these new diabetes medications affects the likelihood of developing cancer in comparison to traditional diabetes medications such as sulfonylureas and metformin.

What did we do?

We searched several medical research databases until November 2023 to find studies about new diabetes medications. We included studies that used real-world patient data and focused on these medications, looking at how often cancer occurred in the patients. To compare the results, we used network meta-analysis to merge the data and estimate the overall risk of cancer associated with these medications.

What did we find?

Our study found that SGLT-2 inhibitors may protect against cancer more than sulfonylureas, GLP-1 agonists, and DPP-4 inhibitors. Additionally, our results showed that DPP-4 inhibitors may lower the risk of developing cancer compared to sulfonylureas.

What do these findings mean?

These findings could impact future clinical practices by guiding healthcare professionals to consider patient safety and cancer prevention when choosing diabetes treatments.

## Introduction

Type 2 diabetes and cancer are significant global health issues, with type 2 diabetes affecting over 400 million people and cancer affecting approximately 20 million people worldwide.^1,2^ An umbrella review of 18 meta-analyses of observational studies has suggested an association between type 2 diabetes and various cancers, including breast, colorectal, liver, pancreatic, and endometrial cancers.^3,4^ Shared risk factors between both conditions, such as smoking, aging, and obesity, contribute to this association.^5^ Furthermore, metabolic mechanisms, such as hyperglycemia and inflammation, are also thought to contribute to this connection.^6,7^ The use of antidiabetic medications may modify the risk of cancer in diabetic patients by impacting the abnormal concentrations of insulin and glucose in the human body, which are essential for cancer development and progression.^6^

In recent years, novel antidiabetic medications, including sodium-glucose cotransporter-2 inhibitors (SGLT-2i), dipeptidyl peptidase-4 inhibitors (DPP-4i), and glucagon-like peptide-1 agonists (GLP-1a), have been increasingly used to treat type 2 diabetes.^8,9^ In addition to their blood sugar-lowering effects, these medications potentially offer additional benefits, such as weight control, blood pressure reduction, and cardio- and renal-protective effects.^10^ However, current evidence regarding the cancer risk or protective effect associated with these novel antidiabetic medications is inconsistent. One international cohort study found no increased risk of pancreatic cancer in users of DPP-4i and GLP-1a compared to users of sulfonylureas (SUs). In contrast, a European cohort study found an increased risk compared to sulfonylureas and metformin users.^11,12^ Additionally, an international cohort study found no association between SGLT-2i and the risk of bladder cancer when compared to DPP-4i and GLP-1a.^13^ These associations differ from the results of previous randomized controlled trials (RCTs) that reported a higher risk of bladder cancer in SGLT-2i users.^14^

Previous meta-analyses have investigated the relationships between various antidiabetic medications and the risk of developing cancer. However, these studies lack direct comparisons among individual drug classes. Additionally, these studies rely on data from RCTs with limitations such as short follow-up periods, small sample sizes, and limited demographic representation, which may reduce their generalizability to real-world scenarios.^15–22^ To our knowledge, only one meta-analysis (MA) based on observational studies has specifically examined the pancreatic cancer risks associated with DPP-4i and GLP-1a.^23^ However, no comprehensive MA of observational studies has yet compared the overall risks or benefits of developing cancer associated with SGLT-2i, DPP-4i, and GLP-1a.

Given the lack of previous comprehensive MA of observational studies, we conducted a comprehensive systematic review and network meta-analysis (NMA) of observational studies to assess the comparative cancer risks or protective effects of novel antidiabetics, compared to other antidiabetics. We hypothesized that novel antidiabetic medications have protective effects against developing cancer compared to other antidiabetics.

## Methods

This NMA was registered in PROSPERO (CRD42023469941) and conducted according to the Preferred Reporting Items for Systematic Reviews and Meta-Analyses (PRISMA) guidelines.^24^ Preliminary findings of the study were presented at the conference for Health Economics and Outcomes Research (ISPOR 2024, Atlanta, GA, USA).^25^

### Search procedures and study selection

**Figure 1.**
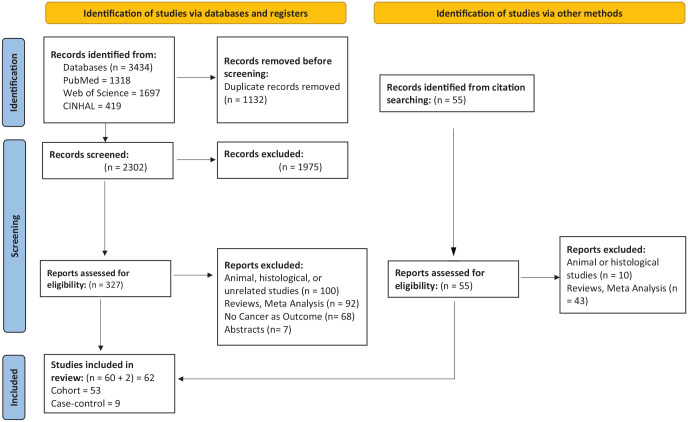
PRISMA 2020 flow diagram. PRISMA, Preferred Reporting Items for Systematic Reviews and Meta-Analyses.

We conducted a comprehensive search of PubMed, Web of Science, and CINAHL databases from their inception until November 30, 2023, with the assistance of a health sciences librarian from The University of Texas at Austin. Our search terms included the generic names of novel antidiabetic medications (SGLT-2i, DPP-4i, and GLP-1a), in addition to various terms for cancer, such as malignancy and tumor (Supplemental Table 1). We also checked the references of the included studies and previous reviews for eligible studies (Figure 1). Our inclusion criteria consisted of comparative observational studies with at least one class of novel antidiabetic medications in the intervention arm, compared to other antidiabetic medications. Other antidiabetic medications included metformin, sulfonylureas, thiazolidinediones (TZDs), insulins, alpha-glucosidase inhibitors, and meglitinides. Additional inclusion criteria were cancer as an outcome and articles published in full-text English. The exclusion criteria were non-human research and other types of studies, including randomized controlled trials (RCTs), case studies, case series, reviews, systematic reviews, and meta-analyses. Abstracts were excluded due to incomplete information. To minimize bias, we excluded studies that aggregated patients receiving different classes of antidiabetic medications in one group and compared them to one group of novel antidiabetic medications. The titles and abstracts were reviewed for eligibility using EndNote^®^ 20.^26^

### Data extraction

After removing duplicates, A.K. and A.A. independently reviewed the remaining studies for eligibility and extracted the following: first author name, publication year, data source, primary race, cancer type, classes of antidiabetics, sample size, mean age, follow-up time, percentage of males, duration of diabetes, hemoglobin A1C (HbA1c), and cancer incidence. A third researcher (G.O.) resolved discrepancies between the extracted information.

### Risk of bias and quality assessment

We used the seven domains of the Risk Of Bias In Non-randomized Studies—of Interventions (ROBINS-I) tool to assess the studies’ risk of bias.^27^ Additionally, the Newcastle–Ottawa Scale was used to assess the quality of included studies, evaluating non-randomized studies (cohort and case-control) on group selection, comparability, and exposure ascertainment.^28^

We assessed the certainty of evidence in our NMA using the Grading of Recommendations, Assessment, Development, and Evaluations (GRADE) approach. The GRADE spreadsheet was used to evaluate the direct, indirect, and network-relative estimates of each comparison in the NMA. The spreadsheet automatically rated each comparison based on the incoherence between the estimates of direct and indirect effects, publication bias, risk of bias, inconsistency, indirectness, and imprecision. We started the quality rating from (low) due to the lack of randomization in observational studies.^29,30^ Publication bias for each direct comparison was assessed using Egger’s test, with a *p* value of 0.05 as the cut-off point.

All assessments (risk of bias, quality assessment, and certainty of evidence) were conducted by different pairs of independent researchers; A.K. and A.A. for risk of bias, A.K. and G.O. for quality assessment, and A.K. and Y.L. for certainty of evidence. Discrepancies between the two assessment grades were resolved by a third researcher (A.A., G.O., or Y.L.).

### Network meta-analysis

To have more homogeneous studies in our NMA, we selected only cohort studies that reported cancer incidence and sample size. A random effects model with informative priors was used to estimate the pooled odds ratio (OR) with 95% credible intervals (CrI) based on the Bayesian framework. This approach was used because the included studies had different patient characteristics, cancers, lag times, follow-up times, and varying prior information about cancer outcomes in previous observational studies and RCTs. A Bayesian framework was adopted because it was an intuitive approach and incorporated prior information into the model.^31^ We provided pooled ORs based on a fixed effects model as a sensitivity analysis. Using a fixed effects model in sensitivity analysis allowed us to assess the similarity between the results of both models, indicating the degree of heterogeneity among the included studies.^32^

The NMA was conducted using the Excel-based interface—NetMetaXL 1.6.1 (Canadian Agency for Drugs and Technologies in Health, Ottawa, ON, Canada), which facilitated the use of the freely available WinBUGS 1.4.3 (Medical Research Council Biostatistics Unit, Cambridge, UK) software within the Excel sheet.^33,34^ This tool used the Markov chain Monte Carlo method to conduct Bayesian-based NMA. NetMetaXL provided readily available sheets for data entry and macros for generating NMA outputs, including network diagrams, probability plots, forest plots with heterogeneity ( $\tau_{\mathrm{new}}^{2}$ ) values, and inconsistency plots. Additionally, league (pairwise comparisons) tables were available within the tool. These tables depended on the surface under the cumulative ranking (SUCRA) to rank the interventions or treatments according to their overall impact on the outcome (cancer risk). NetMetaXL can also incorporate multiple comparisons at the same time, allowing the inclusion of multiple treatment arms from the same study.^35^ Additional NMAs were conducted for site-specific cancers, including gastrointestinal, genitourinary, and breast cancers. The included cancer sites were classified according to the National Cancer Institute classification of cancers.^36^

## Results

### Characteristics of included studies

Our search retrieved 3434 articles, out of which 327 studies were screened for eligibility after initial screening and duplicate removal. Following the eligibility assessment, 267 studies were excluded. Including the results from citation searching, we identified 62 observational studies (53 cohort and 9 case–control) in this systematic review (Figure 1).^11–13,37–95^

Table 1 shows the characteristics of the studies included in the NMA. The mean baseline duration of diabetes in the included studies ranged from 1.18 to 9.05 years, with a follow-up time of 1–5.6 years. The mean baseline age ranged from 51.52 to 75.56 years, with the percentage of males ranging from 38.62% to 73.20%. The predominant races in our systematic review were White (33 studies, 53.23%), Asian (26 studies, 41.94%), and unknown (3 studies, 4.84%). The novel antidiabetic medications included DPP-4i (48 studies, 77.42%), GLP-1a (25 studies, 40.32%), SGLT-2i (13 studies, 20.97%), and incretin-based (both DPP-4i and GLP-1a; 6 studies, 9.68%). Sixteen head-to-head comparisons were provided among the novel antidiabetic medications, while the remaining studies compared these medications with other antidiabetics. More details about the included studies are presented in Supplemental Tables 2 and 3.

**Table 1.** Characteristics of the included studies (overall risk of cancer NMA).*

Author	Data source	Primary race	Cancer type	Class of antidiabetics	Total sample size	Mean age (years)	Mean follow-up time (years)	Male proportion (%)	Mean duration of diabetes (years)	HbA1c (%)
Abrahami et al.^38^	Linked data	White	Colorectal	DPP-4i, GLP-1a, SUs	25,692	66.96	3.50	58.67	4.25	NA
Abrahami et al.^13^	Linked and claims data	White	Bladder	DPP-4i, GLP-1a, SGLT-2i	2,029,802	61.56	1.50–2.60**	53.11	8.24	NA
Bea et al.^39^	Claims data	Asian	Thyroid	DPP-4i, GLP-1a, SGLT-2i	1,365,032	59.00	2.77	58.42	NA	NA
Chan et al.^40^	Linked data	Asian	Colorectal	DPP-4i, SGLT-2i	25,318	60.85	4.95	55.35	1.49	8.10
Choi et al.^42^	EHR	Asian	All	DPP-4i, metformin	1538	63.90	2.20**	56.90	3.10	7.80
Chou et al.^44^	Linked data	Asian	Pancreas	DPP-4i, SGLT-2i	12,958	61.90	NA	58.70	NA	8.35
Chung et al.^45^	Linked data	Asian	All	DPP-4i, SGLT-2i	36,334	58.25	NA	61.35	1.60	8.30
Funch et al.^50^	Claims data	White	Breast	DPP-4i, GLP-1a, SUs, metformin, TZDs	108,182	52**	1.49**	0	NA	NA
Funch et al.^51^	Claims data	White	Thyroid	DPP-4i, GLP-1a, SUs, metformin, TZDs	255,094	53	1.40**	46.95	NA	NA
Garry et al.^54^	Claims data	White	Bladder	DPP-4i, TZDs	91,089	75.51	1.13	39.60	NA	NA
Gokhale et al.^56^	Claims data	White	Pancreas	DPP-4i, TZDs, SUs	137,623	75.56	0.83**	38.62	NA	NA
Hicks et al.^58^	Linked data	White	Breast	DPP-4i, GLP-1a	2920	66.44	3.50	0	7.63	NA
Htoo et al.^60^	Claims data	White	Colorectal	DPP-4i, TZDs, SUs	193,394	75.47	NA	38.77	NA	NA
Kubota et al.^65^	Claims data	Asian	Pancreas	DPP-4i, SUs, metformin, TZDs, SGLT-2i	165,355	53.50	1.30**	73.20	NA	NA
Li et al.^69^	Linked data	Asian	Bladder	SGLT-2i, TZDs	13,906	56.93	2.80	54.30	9.05	7.70
Lu et al.^71^	Linked data	White	Prostate	DPP-4i, GLP-1a, SUs	285,964	59.49	4.58**	100	6.63	NA
Pradhan et al.^74^	Linked data	White	Skin	DPP-4i, SUs	611,117	60.31	2.60^3^	56.81	6.17	NA
Shin and Kim^79^	EHR	Asian	Pancreas	DPP-4i, metformin	4860	56.60	2.90**	57.80	3.53**	7.80
Suissa et al.^81^	Linked data	White	Breast	DPP-4i, SGLT-2i	46,569	NA	2.60	0	NA	NA
Sütő et al.^82^	Claims data	White	All	DPP-4i, SGLT-2i	36,699	NA	1.75	NA	NA	NA
Ueda et al.^89^	Registry	White	Kidney, bladder	GLP-1a, SGLT-2i	213,578	62	2.62**	64	NA	NA
Ueda et al.^90^	Registry	White	Cholangiocarcinoma	DPP-4i, GLP-1a, SUs	585,876	63.74	4.85**	57.89	NA	NA

* Only cohort studies were included in the NMA.

** Median or median range.

DPP-4i, dipeptidyl peptidase-4 inhibitors; EHR, electronic health records; GLP-1a, glucagon-like peptide-1 agonists; NA, not available in the study or unable to calculate due to missing data; NMA, network meta-analysis; SGLT-2i, sodium-glucose cotransporter-2 inhibitors; SUs, sulfonylureas; TZDs, thiazolidinediones.

### Risk of bias and quality assessment

Supplemental Table 4 presents the NMA estimates and their certainty of evidence using GRADE. Egger’s test showed *p* values >0.05 for all direct comparisons with three studies or more, indicating no serious publication bias. The studies with (very low) ratings were mainly affected by their risk of bias and/or the imprecision of the indirect estimates. Most studies (*n* = 58, 93.5%) were considered high quality. The remaining studies had poor assessment scores because they used pharmacovigilance databases without appropriate comparative groups or adequate control of confounders (Supplemental Table 5). Half (50%) of the studies had a low or medium risk of bias. Insufficient control over crucial confounding factors, such as smoking and alcohol consumption, resulted in the downgrading of assessments for other studies (Supplemental Table 6).

### Overall risk of cancer NMA

**Figure 2.**
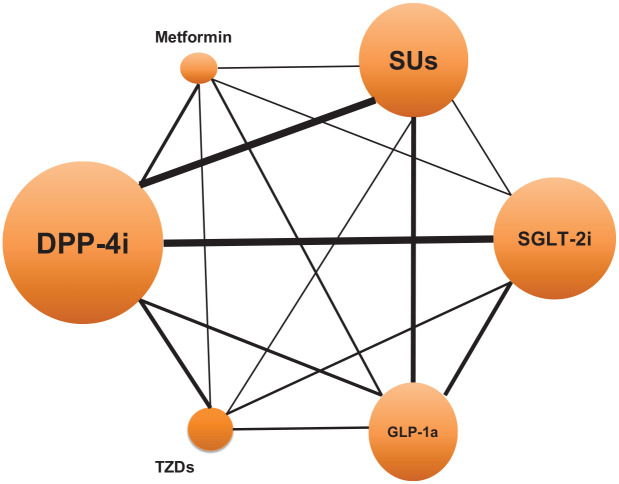
Network diagram for the overall risk of cancer NMA. DPP-4i, dipeptidyl peptidase-4 inhibitors; GLP-1a, glucagon-like peptide-1 agonists; NMA, network meta-analysis; SGLT-2i, sodium-glucose cotransporter-2 inhibitors; SUs, sulfonylureas; TZDs, thiazolidinediones.

The NMA included 22 studies (37 comparisons) with a total of 6,041,368 patients and 24,017 cancer cases. These studies compared novel antidiabetic medications with metformin, sulfonylureas, and TZDs. Figure 2 shows the network of comparisons for the outcome assessed in our NMA, and Supplemental Table 7 presents the number of cancer events for each comparison.

**Figure 3.**
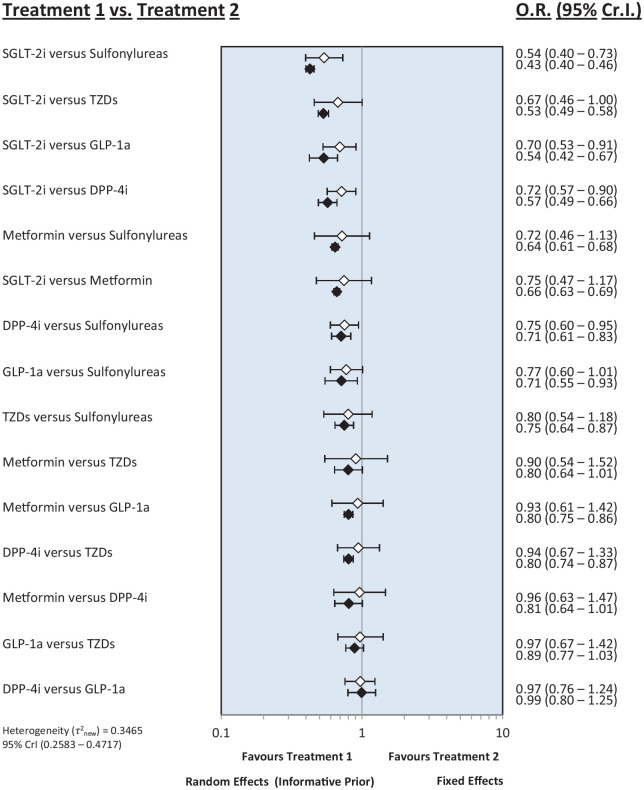
Forest plot of the overall risk of cancer for all pairwise class comparisons. DPP-4i, dipeptidyl peptidase-4 inhibitors; GLP-1a, glucagon-like peptide-1 agonists; SGLT-2i, sodium-glucose cotransporter-2 inhibitors; TZDs, thiazolidinediones.

Figure 3 shows the overall risk of cancer for all pairwise class comparisons. SGLT-2i were found to be more effective in reducing the overall risk of cancer when compared to sulfonylureas (OR: 0.54; 95% CrI: 0.40–0.73, low certainty), GLP-1a (OR: 0.70; 95% CrI: 0.53–0.91, low certainty), and DPP-4i (OR: 0.72; 95% CrI: 0.57–0.90, very low certainty). However, no significant results were observed when comparing SGLT-2i with metformin (OR: 0.75; 95% CrI: 0.47–1.17, very low certainty) and TZDs (OR: 0.67; 95% CrI: 0.46–1, very low certainty). DPP-4i were found to be significantly associated with a lower risk of cancer compared to sulfonylureas (OR: 0.75; 95% CrI: 0.60–0.95, low certainty). No statistically significant ORs were observed in other head-to-head comparisons. The fixed effects model yielded similar results to the random effects model, indicating low heterogeneity among the included studies.

When ranking antidiabetic medications based on their effectiveness in reducing the overall risk of cancer, SGLT-2i had the highest probability of being the safest (SUCRA = 0.97), followed by metformin (SUCRA = 0.58) and DPP-4i (SUCRA = 0.53). Sulfonylureas had the lowest probability of being the safest, with a SUCRA score of 0.05 (Supplemental Figures 1 and 2). After visually inspecting the inconsistency plot of the NMA, no potential inconsistency was detected (Supplemental Figure 3).

### Site-specific cancer risk NMA

#### Gastrointestinal cancer

**Figure 4.**
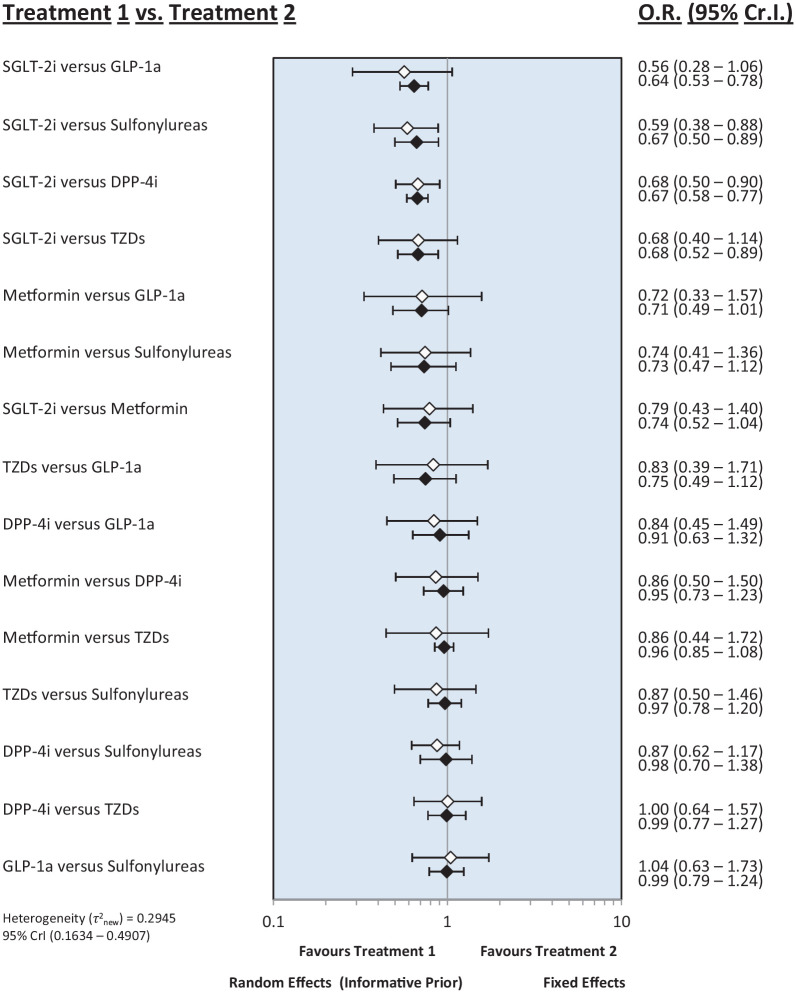
Forest plot of the risk of gastrointestinal cancer. DPP-4i, dipeptidyl peptidase-4 inhibitors; GLP-1a, glucagon-like peptide-1 agonists; SGLT-2i, sodium-glucose cotransporter-2 inhibitors; TZDs, thiazolidinediones.

Figure 4 shows the risk of gastrointestinal cancer for all pairwise class comparisons. The risk of gastrointestinal cancer included 11 studies with 17 comparisons. SGLT-2i were found to significantly reduce the risk of gastrointestinal cancers compared to sulfonylureas (OR: 0.59; 95% CrI: 0.38–0.88) and DPP-4i (OR: 0.68; 95% CrI: 0.50–0.90). No statistically significant ORs were observed in the other comparisons. SGLT-2i had the highest probability of being the safest intervention to reduce the risk of gastrointestinal cancers (SUCRA = 0.94), followed by metformin (SUCRA = 0.65) and DPP-4i (SUCRA = 0.55). Sulfonylureas had the lowest probability of ranking safest, with a SUCRA score of 0.24.

#### Genitourinary cancer

Supplemental Figure 4 presents the risk of genitourinary cancer for all pairwise class comparisons. SGLT-2i were associated with a significantly lower risk of genitourinary cancers when compared to DPP-4i (OR: 0.53; 95% CrI: 0.45–0.61), TZDs (OR: 0.34; 95% CrI: 0.24–0.46), and sulfonylureas (OR: 0.31; 95% CrI: 0.25–0.39). GLP-1a also had a significantly lower risk of genitourinary cancers compared to DPP-4i (OR: 0.57; 95% CrI: 0.46–0.69), TZDs (OR: 0.36; 95% CrI: 0.26–0.52), and sulfonylureas (OR: 0.34; 95% CrI: 0.26–0.43). In addition, DPP-4i demonstrated a lower risk of genitourinary cancers compared to TZDs (OR: 0.64; 95% CrI: 0.48–0.85) and sulfonylureas (OR: 0.59; 95% CrI: 0.49–0.71). SGLT-2i continued to show the highest probability of being ranked first for reducing the risk of genitourinary cancers (SUCRA = 0.97), followed by GLP-1a (SUCRA = 0.78) and DPP-4i (SUCRA = 0.50). Sulfonylureas had the lowest probability of being ranked first, with a SUCRA score of 0.08. This analysis consisted of 7 studies and 12 comparisons from different genitourinary sites, including the bladder, prostate, and kidney.

#### Breast cancer

Regarding breast cancer risk, we included six studies with nine comparisons. SGLT-2i demonstrated a significantly lower risk of breast cancer compared to DPP-4i (OR: 0.63; 95% CrI: 0.46–0.87), metformin (OR: 0.48; 95% CrI: 0.23–0.98), sulfonylureas (OR: 0.42; 95% CrI: 0.20–0.89), and GLP-1a (OR: 0.43; 95% CrI: 0.26–0.72). SGLT-2i also had the highest SUCRA probability (0.98), followed by DPP-4i (SUCRA = 0.67) and TZDs (SUCRA = 0.54). GLP-1a had the lowest probability of reducing the risk of breast cancer, with a SUCRA score of 0.22 (Supplemental Figure 5). More detailed information about the number of site-specific cancer events within each comparison is available in Supplemental Table 8.

## Discussion

We conducted a thorough systematic review and NMA to evaluate the relationship between different classes of antidiabetics and their potential cancer risks. The NMA included 6,041,368 diabetic patients with 24,017 cancer events. Our study indicated that SGLT-2i were significantly associated with a reduced overall risk of cancer compared to sulfonylureas, GLP-1a, and DPP-4i. Additionally, DPP-4i demonstrated a significantly lower overall cancer risk compared to sulfonylureas. Analysis of site-specific cancer risks revealed that SGLT-2i had a protective effect against gastrointestinal, genitourinary, and breast cancers compared to other antidiabetics.

The observed protective effect of SGLT-2i aligns with histological evidence reporting the impact of SGLT-2i on eliminating tumor cells.^96^ This reduction in tumor cells may be explained by the ability of SGLT-2i to lower glucose uptake in various cancer models, decrease inflammation biomarkers, and reduce cancer cell proliferation.^96,97^ Mechanisms behind the cancer risk reduction of SGLT-2i have been discussed in many studies. Zhou et al. and Villani et al. reported that dapagliflozin and canagliflozin inhibited the growth of breast cancer cells by decreasing their proliferation and enhancing the activity of 5′ adenosine monophosphate-activated protein kinase (AMPK). AMPK can inhibit the progression of cancer cells by the regulation of inflammatory pathways.^98,99^ Additionally, research by Nakano et al. and Xu et al. showed that canagliflozin inhibited the growth of hepatocellular and pancreatic cancer cells, respectively.^100,101^ Other studies also reported the inhibitory effect of SGLT-2i on osteosarcoma and thyroid cancer cells.^96^ These findings suggest that SGLT-2i may play a significant role in cancer progression by targeting multiple pathways involved in tumor growth.

Contrary to our results, previous meta-analyses did not show a cancer protective effect of SGLT-2i.^19–21,102^ An overview of quantitative systematic reviews by Pelletier et al., which included RCTs and observational studies, found no difference in the cancer protective effects between the users of SGLT-2i and non-users (point estimate range 0.72–1.42). The results of site-specific cancers in Pelletier et al. were non-significant except for empagliflozin and canagliflozin. In their analysis, empagliflozin was associated with an increased risk of bladder cancer, while canagliflozin appeared to be protective for gastrointestinal cancer.^102^ An MA of 46 RCTs with a mean follow-up period of 61 weeks showed that SGLT-2i were not significantly associated with an increased risk of overall cancer compared to placebo or other antidiabetics (OR: 1.14; 95% CI: 0.96–1.36). Interestingly, this review also reported an increased risk of bladder cancer with empagliflozin use (OR: 4.49; 95% CI: 1.21–16.73) and a lower risk of gastrointestinal cancer with canagliflozin use (OR: 0.15; 95% CI: 0.04–0.60).^14^ Our results may differ from those of previous reviews based on RCTs, potentially due to the limited duration of follow-up in these studies. Rokszin et al. showed that SGLT-2i had a late risk reduction in some types of cancer, including breast, lower gastrointestinal tract, and lung cancers.^75^ In addition, reviews of RCTs included other users of SGLT-2, including patients with heart failure and chronic kidney disease. Those patients do not share the same risk factors for cancer as diabetic patients included in our study. These risk factors include hyperglycemia, insulin resistance, inflammation, and diabetes-related comorbidities.^6,7^

In our analysis, metformin ranked second among the antidiabetic medications in terms of reducing the overall risk of cancer with a SUCRA score of 0.58. This finding is consistent with a previous MA that demonstrated an association between metformin use and a lower overall risk of cancer in diabetic patients (Relative Risk (RR): 0.61; 95% CI: 0.54–0.70).^103^ Compared to other antidiabetic medications, metformin was also found to be associated with a lower risk of some site-specific cancers, including thyroid (OR: 0.68; 95% CI: 0.50–0.91), esophageal (Hazard Ratio (HR): 0.69; 95% CI: 0.54–0.87), and pancreatic (OR: 0.82; 95% CI: 0.69–0.98) cancers.^104–106^ These findings are supported by preclinical studies, which have demonstrated that metformin exhibits inhibitory effects on cancer cells. These effects are thought to be mediated through the activation of AMPK, reduction of hyperlipidemia, inhibition of the cancer cell cycle, and enhancement of immune system activity.^107^

Recently, the cancer risk of incretin-based antidiabetics (DPP4i and GLP-1a) has gained attention due to their increased use in diabetes and weight control.^108^ In our NMA, DPP-4i ranked third with a significant cancer protective effect compared to sulfonylureas. An MA of 115 RCTs showed a decreased overall risk of cancer in DPP-4i users compared to non-users (OR: 0.91; 95% CI: 0.80–0.97), with a significant decrease in the risk of rectal and skin cancers.^109^ Moreover, the use of DPP-4i in the MA of Overbeek et al. had a lower risk of breast cancer (HR: 0.76; 95% CI: 0.60–0.96); however, it had no association with the risk of overall cancer.^110^ The mechanism by which DPP-4i decrease cancer risk remains unclear. Almagthali et al. suggested that DPP-4i have an anticancer effect by increasing the number of immune cells within the cancer area.^111^ However, Busek et al. stated that this effect may be beneficial only in selected cancer types like ovarian and liver cancers.^112^ Unlike DPP-4i, GLP-1a did not show a cancer protective effect in previous meta-analyses.^15,18,113–117^ However, two recent studies by Wang et al. suggested that GLP-1a may have cancer protective effects in diabetic patients with more than 15 years of follow-up.^118,119^ The first study found that, compared to insulin, GLP-1a may decrease the risk of certain obesity-associated cancers, such as kidney, ovarian, multiple myeloma, colorectal, and ovarian cancers.^118^ The second study showed that GLP-1a use was associated with reduced colorectal cancer risk compared to other antidiabetics, such as insulin, metformin, SGLT-2i, TZDs, and sulfonylureas.^119^ The long-term weight loss effect of GLP-1a may explain the results of these two studies. Obesity is a known risk factor for cancer, with almost 6% of all cancers being linked to obesity.^120^ Previous studies demonstrated that the inflammatory cytokines produced by adipose tissue could potentially contribute to cancer development.^121,122^ For that, the use of GLP-1a, along with a healthy lifestyle, may play a significant role in cancer prevention. The potential cancer protective effects of GLP-1a, particularly against obesity-associated cancers, stand in contrast to concerns raised by the conflicting results regarding thyroid cancer. A recent MA of 64 RCTs showed a moderate risk of thyroid cancer associated with the use of GLP-1a compared to other antidiabetics (OR: 1.52; 95% CI: 1.01–2.29).^123^ An older analysis by Hu et al. did not find a significant relationship between thyroid cancer and the use of GLP-1a.^114^ The current carcinogenic studies suggest that GLP-1a may stimulate the proliferation of thyroid cells, potentially increasing the risk of thyroid cancer.^115^ Given the conflicting results, additional studies are needed to clarify the relationship between GLP-1a and thyroid cancer.

While the use of TZDs had no association with the risk of overall cancer, the reports regarding their risk of site-specific cancers are inconsistent.^124^ A safety review by the US Food and Drug Administration concluded that the use of TZDs, namely pioglitazone, may increase the risk of bladder cancer.^125^ This review was supported by multiple meta-analyses of observational studies and RCTs.^126–128^ At the same time, TZDs were reported to have a protective effect against colorectal (RR: 0.91; 95% CI: 0.84–0.99) and breast (RR: 0.81; 95% CI: 0.66–0.99) cancers in observational studies.^129,130^ The underlying mechanism behind the increased risk of bladder cancer among TZD users is not fully understood. However, their protective effect in other cancers may be explained by the activation of the peroxisome proliferator-activated receptor gamma (PPAR-γ). The activation of PPAR-γ has anti-cancer effects, including growth inhibition and apoptosis.^131^

With a SUCRA score of 0.05, sulfonylureas had the lowest probability of being the safest in our main analysis. The high risk of cancer associated with sulfonylureas in our analysis aligns with the MA of cohort studies conducted by Thakkar et al. (RR: 1.55; 95% CI: 1.48–1.63).^132^ The observed cancer risk may be attributed to the effect of sulfonylureas on insulin levels, as these drugs stimulate the pancreas to release insulin.^133^ Insulin can increase cancer risk through several mechanisms. These mechanisms include promoting cancer cell proliferation and protecting cells from apoptosis. Hyperinsulinemia also increases free bioactive insulin-like growth factor-I levels, which have a strong mitogenic effect on cancer cells. Additionally, hyperinsulinemia indirectly raises the levels of sex steroids, leading to an increase in the risk of postmenopausal breast and endometrial cancers.^121^ The insulin effect on cancer progression may explain the increased risk of cancer in antidiabetics that elevate insulin levels, such as sulfonylureas, compared to other antidiabetic treatments like GLP-1a and SGLT-2i, which do not raise insulin to the same extent.^134,135^ High glycemic index foods, which contribute to elevated insulin levels, have been associated with an increased risk of diabetes-related cancers. This supports the argument that increased insulin levels could be a significant factor in cancer risk in antidiabetics that increase insulin levels.^136,137^ Interestingly, Thakkar et al. reported no relationship between sulfonylureas and cancer risk in RCTs (RR: 1.17; 95% CI: 0.95–1.45) or case–control studies (OR: 1.02; 95% CI: 0.93–1.13).^132^ This inconsistency is likely due to the diverse pharmacological agents within the sulfonylurea class. Each agent has its own unique receptor affinity, systemic exposure, and anticancer activity.^138^ Monami et al. and Yang et al. supported that gliclazide users had a lower cancer risk compared to non-users.^139,140^ Conversely, additional studies showed that glibenclamide had a higher risk of cancer compared to non-users or users of other sulfonylureas.^139,141,142^ These observations necessitate assessing the agents of sulfonylureas independently to prevent biased conclusions about sulfonylureas in general.

## Limitations

There are several limitations in our study. First, we included certain observational studies in our NMA (22 studies, 35.48%) because they provided case numbers and sample sizes. We also excluded studies that compared novel antidiabetics with study groups comprising patients receiving multiple classes of antidiabetics. This step was crucial to reduce the heterogeneity between the included studies and reduce the bias associated with including patients receiving multiple antidiabetics. Second, the main analysis included varying cancer types with different sample sizes and follow-up durations, which could introduce heterogeneity into the overall evidence. To address this limitation, a sensitivity analysis was conducted using a fixed-effect model to ensure the robustness of our main findings. The results from the fixed-effect model were consistent with the results of the random effects model, suggesting low heterogeneity. Noting that the heterogeneity ( $\tau_{\mathrm{new}}^{2}$ ) values ranged from 0.08 to 0.35 in the overall and site-specific risk of cancers NMA, suggesting low to moderate heterogeneity among the included studies.^143^ Additionally, some studies in this review had low bias and quality assessments because of insufficient control over important confounders and the absence of appropriate comparators. We addressed this by using the GRADE approach to assess the certainty of evidence in our NMA. GRADE is a systematic and clear method for evaluating the certainty of a body of evidence by providing well-defined key questions.^28^ Finally, our NMA did not include insulin because only one study with insulin as a comparator met our inclusion criteria.^93^ Including a single study would not have provided conclusive evidence about insulin in this NMA. Because of insulin’s potential role as a growth factor and its relationship with cancer risk, further studies with a larger number of insulin comparisons are needed to assess its impact on cancer risk relative to novel antidiabetic medications.

Despite these limitations, our study has several strengths. To our knowledge, this is the first study to synthesize granular data on cancer incidence among patients utilizing novel antidiabetic medications and direct comparisons among them. We have synthesized evidence from observational studies across various cancer types, offering a comprehensive overview of the association between antidiabetics and cancer risk. Our findings could have significant implications for clinical practice by assisting healthcare providers in selecting antidiabetic medications with potential cancer protective effects for patients at higher risk of cancer. Future research should prioritize studying individual antidiabetic agents, while also conducting direct comparisons among them. Focusing on each agent individually avoids biased conclusions about antidiabetic drug classes as a whole.

## Conclusion

The current NMA, based on observational studies, shows that SGLT-2i are associated with a lower risk of developing cancer compared to sulfonylureas, GLP-1a, and DPP-4i. These results may guide future clinical practice by considering medication selections in diabetic patients, focusing on patient safety and cancer protection. Future studies should focus on head-to-head comparisons among antidiabetics, with further research to explore the mechanisms behind these associations.

## Supplemental Material

sj-docx-1-taw-10.1177_20420986251335214 – Supplemental material for Real-world evidence on the association of novel antidiabetic medication use with cancer risk and protective effects: a systematic review and network meta-analysis Supplemental material, sj-docx-1-taw-10.1177_20420986251335214 for Real-world evidence on the association of novel antidiabetic medication use with cancer risk and protective effects: a systematic review and network meta-analysis by Ahmed S. Kenawy, Yi-Shao Liu, Ayobami Aiyeolemi, Godwin Okoye and Chanhyun Park in Therapeutic Advances in Drug Safety

sj-docx-2-taw-10.1177_20420986251335214 – Supplemental material for Real-world evidence on the association of novel antidiabetic medication use with cancer risk and protective effects: a systematic review and network meta-analysis Supplemental material, sj-docx-2-taw-10.1177_20420986251335214 for Real-world evidence on the association of novel antidiabetic medication use with cancer risk and protective effects: a systematic review and network meta-analysis by Ahmed S. Kenawy, Yi-Shao Liu, Ayobami Aiyeolemi, Godwin Okoye and Chanhyun Park in Therapeutic Advances in Drug Safety

## Declarations

**Ethics approval and consent to participate:** Not applicable. Our study is a systematic review and NMA of the literature, with no participants.

**Consent for publication:** Not applicable.

**Availability of data and materials:** All the extracted data are available in Table 1, Supplemental Tables 3, 7, and 8.
