# Supplementary material for: Real-world evidence on the association of novel antidiabetic medication use with cancer risk and protective effects: a systematic review and network meta-analysis
Source: Ther Adv Drug Saf. 2025 Apr 21;16:20420986251335214. doi: 10.1177/20420986251335214 (PMC12033536; doi:10.1177/20420986251335214)
Supplement: sj-docx-2-taw-10.1177_20420986251335214 – Supplemental material for Real-world evidence on the association of novel antidiabetic medication use with cancer risk and protective effects: a systematic review and network meta-analysis [file sj-docx-2-taw-10.1177_20420986251335214.docx]

**Real-World Evidence on the Association of Novel Antidiabetic Medication Use with Cancer Risk and Protective Effects: A Systematic Review and Network Meta-Analysis**

**Supplementary Materials**

**Supplementary Tables:**

Supplementary Table 1. Search terms used for systematic review article identification (Date: Nov. 30, 2023).

Supplementary Table 2. Types and frequencies of cancer comparisons.

Supplementary Table 3. Characteristics of the studies included in the systematic review (not included in the NMA).

Supplementary Table 4. Certainty of evidence (GRADE: Grading of Recommendations Assessment, Development and Evaluation).

Supplementary Table 5. Quality assessment (Newcastle Ottawa Scale).

Supplementary Table 6. Risk of bias (ROBINS-I: Risk Of Bias In Non-randomised Studies - of Interventions).

Supplementary Table 7. The number of cancer events within each comparison (overall risk of cancer).

Supplementary Table 8. The number of site-specific cancer events within each comparison.

**Supplementary Figures:**

Supplementary Figure 1. League table of the overall risk of cancer for all pairwise class comparisons.

Supplementary Figure 2. Rankogram and SUCRA scores for the overall risk of cancer NMA.

Supplementary Figure 3. Inconsistency graph for the overall risk of cancer NMA.

Supplementary Figure 4. Forest plot for risk of genitourinary cancer NMA.

Supplementary Figure 5. Forest plot for risk of breast cancer NMA.

**Supplementary Table 1. Search terms used for systematic review article identification (Date: Nov. 30, 2023).**

| **Database** | **Search terms** | **Outcome** |
| --- | --- | --- |
| **PubMed** | ((("dulaglutide" [Supplementary Concept] OR "Exenatide"[Mesh] OR "semaglutide" [Supplementary Concept] OR "Liraglutide"[Mesh] OR "lixisenatide" [Supplementary Concept] OR "Glucagon-Like Peptides"[Mesh] OR "empagliflozin" [Supplementary Concept] OR "Canagliflozin"[Mesh] OR "dapagliflozin" [Supplementary Concept] OR "ertugliflozin" [Supplementary Concept] OR "bexagliflozin" [Supplementary Concept] OR "Sodium-Glucose Transporter 2 Inhibitors"[Mesh] OR "Sodium-Glucose Transporter 2 Inhibitors" [Pharmacological Action] OR "Sitagliptin Phosphate"[Mesh] OR "saxagliptin" [Supplementary Concept] OR "Linagliptin"[Mesh] OR "alogliptin" [Supplementary Concept] OR "Vildagliptin"[Mesh] OR "Dipeptidyl-Peptidase IV Inhibitors"[Mesh] OR "Dipeptidyl-Peptidase IV Inhibitors" [Pharmacological Action] OR dulaglutide[Title/Abstract] OR exenatide[Title/Abstract] OR semaglutide[Title/Abstract] OR liraglutide[Title/Abstract] OR lixisenatide[Title/Abstract] OR "glucagon-like peptide*"[Title/Abstract] OR "GLP 1 agonist*"[Title/Abstract] OR GLP-1[Title/Abstract] OR "GLP 1"[Title/Abstract] OR empagliflozin[Title/Abstract] OR canagliflozin[Title/Abstract] OR dapagliflozin[Title/Abstract] OR ertugliflozin[Title/Abstract] OR bexagliflozin[Title/Abstract] OR "sodium-glucose transporter 2 inhibitor*"[Title/Abstract] OR SGLT-2[Title/Abstract] OR "SGLT 2"[Title/Abstract] OR sitagliptin[Title/Abstract] OR saxagliptin[Title/Abstract] OR linagliptin[Title/Abstract] OR alogliptin[Title/Abstract] OR vildagliptin[Title/Abstract] OR "dipeptidyl-peptidase IV inhibitor*"[Title/Abstract] OR "DPP-4 inhibitor*"[Title/Abstract])) AND (("Neoplasms"[Mesh] OR "Carcinoma"[Mesh] OR cancer*[Title/Abstract] OR tumor*[Title/Abstract] OR tumour*[Title/Abstract] OR carcinoma*[Title/Abstract] OR neoplasm*[Title/Abstract]))) | 1318 |
| **CINAHL** | ((((MW dulaglutide) OR (MH Exenatide+) OR (MW semaglutide) OR (MH Liraglutide+) OR (MW lixisenatide) OR (MH "Glucagon-Like Peptides+") OR (MW empagliflozin) OR (MH Canagliflozin+) OR (MW dapagliflozin) OR (MW ertugliflozin) OR (MW bexagliflozin) OR (MH "Sodium-Glucose Transporter 2 Inhibitors+") OR "Sodium-Glucose Transporter 2 Inhibitors" OR (MH "Sitagliptin Phosphate+") OR (MW saxagliptin) OR (MH Linagliptin+) OR (MW alogliptin) OR (MH Vildagliptin+) OR (MH "Dipeptidyl-Peptidase IV Inhibitors+") OR "Dipeptidyl-Peptidase IV Inhibitors" OR (TI dulaglutide OR AB dulaglutide) OR (TI exenatide OR AB exenatide) OR (TI semaglutide OR AB semaglutide) OR (TI liraglutide OR AB liraglutide) OR (TI lixisenatide OR AB lixisenatide) OR (TI "glucagon-like peptide*" OR AB "glucagon-like peptide*") OR (TI "GLP 1 agonist*" OR AB "GLP 1 agonist*") OR (TI GLP-1 OR AB GLP-1) OR (TI "GLP 1" OR AB "GLP 1") OR (TI empagliflozin OR AB empagliflozin) OR (TI canagliflozin OR AB canagliflozin) OR (TI dapagliflozin OR AB dapagliflozin) OR (TI ertugliflozin OR AB ertugliflozin) OR (TI bexagliflozin OR AB bexagliflozin) OR (TI "sodium-glucose transporter 2 inhibitor*" OR AB "sodium-glucose transporter 2 inhibitor*") OR (TI SGLT-2 OR AB SGLT-2) OR (TI "SGLT 2" OR AB "SGLT 2") OR (TI sitagliptin OR AB sitagliptin) OR (TI saxagliptin OR AB saxagliptin) OR (TI linagliptin OR AB linagliptin) OR (TI alogliptin OR AB alogliptin) OR (TI vildagliptin OR AB vildagliptin) OR (TI "dipeptidyl-peptidase IV inhibitor*" OR AB "dipeptidyl-peptidase IV inhibitor*") OR (TI "DPP-4 inhibitor*" OR AB "DPP-4 inhibitor*"))) AND (((MH Neoplasms+) OR (MH Carcinoma+) OR (TI cancer* OR AB cancer*) OR (TI tumor* OR AB tumor*) OR (TI tumour* OR AB tumour*) OR (TI carcinoma* OR AB carcinoma*) OR (TI neoplasm* OR AB neoplasm*)))) | 419 |
| **Web of Science** | (((ALL=dulaglutide OR ALL=Exenatide OR ALL=semaglutide OR ALL=Liraglutide OR ALL=lixisenatide OR ALL="Glucagon-Like Peptides" OR ALL=empagliflozin OR ALL=Canagliflozin OR ALL=dapagliflozin OR ALL=ertugliflozin OR ALL=bexagliflozin OR ALL="Sodium-Glucose Transporter 2 Inhibitors" OR ALL="Sodium-Glucose Transporter 2 Inhibitors" OR ALL="Sitagliptin Phosphate" OR ALL=saxagliptin OR ALL=Linagliptin OR ALL=alogliptin OR ALL=Vildagliptin OR ALL="Dipeptidyl-Peptidase IV Inhibitors" OR ALL="Dipeptidyl-Peptidase IV Inhibitors" ))) (((TI=dulaglutide OR AB=dulaglutide) OR (TI=exenatide OR AB=exenatide) OR (TI=semaglutide OR AB=semaglutide) OR (TI=liraglutide OR AB=liraglutide) OR (TI=lixisenatide OR AB=lixisenatide) OR (TI="glucagon-like peptide*" OR AB="glucagon-like peptide*") OR (TI="GLP 1 agonist*" OR AB="GLP 1 agonist*") OR (TI=GLP-1 OR AB=GLP-1) OR (TI="GLP 1" OR AB="GLP 1") OR (TI=empagliflozin OR AB=empagliflozin) OR (TI=canagliflozin OR AB=canagliflozin) OR (TI=dapagliflozin OR AB=dapagliflozin) OR (TI=ertugliflozin OR AB=ertugliflozin) OR (TI=bexagliflozin OR AB=bexagliflozin) OR (TI="sodium-glucose transporter 2 inhibitor*" OR AB="sodium-glucose transporter 2 inhibitor*") OR (TI=SGLT-2 OR AB=SGLT-2) OR (TI="SGLT 2" OR AB="SGLT 2") OR (TI=sitagliptin OR AB=sitagliptin) OR (TI=saxagliptin OR AB=saxagliptin) OR (TI=linagliptin OR AB=linagliptin) OR (TI=alogliptin OR AB=alogliptin) OR (TI=vildagliptin OR AB=vildagliptin) OR (TI="dipeptidyl-peptidase IV inhibitor*" OR AB="dipeptidyl-peptidase IV inhibitor*") OR (TI="DPP-4 inhibitor*" OR AB="DPP-4 inhibitor*"))) (((( ALL=Neoplasms OR ALL=Carcinoma OR (TI=cancer* OR AB=cancer*) OR (TI=tumor* OR AB=tumor*) OR (TI=tumour* OR AB=tumour*) OR (TI=carcinoma* OR AB=carcinoma*) OR (TI=neoplasm* OR AB=neoplasm*))))) | 1697 |

SGLT-2i: sodium-glucose cotransporter-2 inhibitors, DPP-4i: dipeptidyl peptidase-4 inhibitors, GLP-1a: glucagon-like peptide-1 agonists.

**Supplementary Table 2. Types and frequencies of cancer comparisons.**

| **Type of cancer** | **Frequency of comparisons** |
| --- | --- |
| **All cancer types** | **11** |
| **Breast** | **21** |
| **Digestive/Gastrointestinal**  Cholangiocarcinoma  Colon  Colorectal  Digestive organs  Esophagus  Esophagus, stomach, and small intestine  Gallbladder and extrahepatic bile duct  Gastrointestinal (GI)  Liver  Lower GI  Other digestive organs  Pancreas  Rectum  Rectum/Anus  Stomach | **111**  6  5  11  2  2  2  2  1  12  1  1  59  2  2  3 |
| **Genitourinary**  Bladder  Bladder and Kidney  Genitourinary  Kidney  Prostate  Urinary tract  Urothelial  Uterine | **36**  17  1  1  5  7  3  1  1 |
| **Gynecologic**  Endometrial  Ovary | **3**  1  2 |
| **Head and Neck**  Lip, oral cavity, and pharynx  Nasopharynx  Oral cavity  Thyroid  Tongue | **24**  2  1  2  18  1 |
| **Hematologic/Blood**  Hematologic  Leukemia  Lymphatic and hematopoietic tissue  Lymphoma  Multiple myeloma | **9**  2  2  2  2  1 |
| **Musculoskeletal**  Bone | **1**  1 |
| **Neurologic**  Meningioma | **1**  1 |
| **Respiratory/Thoracic**  Lung  Lung and Larynx  Respiratory system  Thymus | **19**  15  1  2  1 |
| **Skin**  Melanoma  Non-Melanoma  Skin | **5**  2  2  1 |
| **Unclassified** | **7** |
| **Total comparisons** | **248** |

**Supplementary Table 3. Characteristics of the studies included in the systematic review (not included in the NMA) (n = 40).**

| **Author** | **Data source** | **Primary race** | **Cancer type** | **Class of antidiabetics** | **Total sample size** | **Mean age (Years)** | **Mean follow-up time (Years)** | **Male proportion (%)** | **Mean duration of diabetes (Years)** | **HbA1c (%)** |
| --- | --- | --- | --- | --- | --- | --- | --- | --- | --- | --- |
| **Cohort studies** | | | | | | | | | | |
| Abrahami et al. 2018^37^ | Linked Data | White | Cholangiocarcinoma | DPP-4i, GLP-1a, other antidiabetics | 53,632 | 66.26 | 2.743 | 58.86 | 8.90 | NA2 |
| Boniol et al. 2018^12^ | Claims data | White | Pancreas | DPP-4i, GLP-1a, other antidiabetics | 559,025 | NA | NA | 49.51 | NA | NA |
| Chen et al. 2023^41^ | Claims data | Asian | Liver | DPP-4i, other antidiabetics | 11,028 | 51.52 | NA | 65.80 | NA | NA |
| Dore et al. 2012^46^ | Claims data | White | Pancreas, Thyroid | GLP-1a, other antidiabetics | 65788 | NA | 1 | 45.30 | NA | NA |
| Elashoff et al. 2011^47^ | Others4 | NA | Any type, Pancreas, Thyroid | DPP-4i, GLP-1a, other antidiabetics | 2,417 | NA | NA | NA | NA | NA |
| Funch et al. 2014^49^ | Claims data | White | Pancreas | DPP-4i, GLP-1a, SUs, Metformin, TZDs | NA | NA | 1.303 | 48.15 | NA | NA |
| Funch et al. 2019^52^ | Claims data | White | Pancreas | DPP-4i, GLP-1a, SUs, Metformin, TZDs | 70,326 | 52 | 1.403 | 46.80 | NA | NA |
| Goossens et al. 2015^57^ | Linked data | White | Bladder | DPP-4i, SUs | NA | NA | NA | NA | NA | NA |
| Hsu et al. 2021^59^ | Claims data | Asian | Liver | DPP-4i, other antidiabetics | 2,166 | 59.95 | NA | 50.90 | NA | NA |
| Hu et al. 2023^61^ | Claims data | Asian | Any type | SGLT-2i, other antidiabetics | 651,979 | 58.56 | 1.55 | 57.80 | NA | NA |
| Karp et al. 2019^62^ | Linked data | White | Any type | DPP-4i, GLP-1a, SUs | 55,814 | 62.90 | 5.603 | 59.43 | 4.42 | 8.73 |
| Kim et al. 2023^63^ | Claims data | Asian | Pancreas | DPP-4i, other antidiabetics | 102,964 | 58 | 83 | 58.55 | NA | NA |
| Knappen et al. 2016^64^ | Linked data | White | Pancreas | DPP-4i, GLP-1a, other antidiabetics | 210,798 | 61.82 | 3.41 | 53.36 | NA | NA |
| Lee et al. 2018^67^ | Claims data | Asian | Pancreas | DPP-4i, other antidiabetics | 966,453 | NA | NA | NA | 3.87 | NA |
| Lee et al. 2019^68^ | Claims data | Asian | Pancreas | DPP-4i, other antidiabetics | 33,208 | 62.04 | 3.46 | 57.80 | NA | NA |
| Liang et al. 2019^70^ | Claims data | White | Pancreas, Thyroid | GLP-1a, other antidiabetics | 82,946 | NA | 1.14 | 48.74 | NA | NA |
| Montvida et al. 2019^72^ | EHR5 | White | Pancreas | DPP-4i, GLP-1a | 62,749 | 56.99 | 2.54 | 45.18 | 1.18 | 7.62 |
| Na et al. 2022^73^ | Claims data | Asian | Lung, Liver, Pancreas, Kidney | DPP-4i, SUs, TZDs, other antidiabetics | 854,413 | 57.21 | NA | 60.96 | 6.82 | NA |
| Rokszin et al. 2021^75^ | Claims data | White | Multiple cancer sites | SGLT-2i, DPP-4i | 322,761 | 59.70 | 1.85 | 52 | 5.15 | NA |
| Romley et al. 2012^76^ | Claims data | White | Pancreas | GLP-1a, other antidiabetics | NA | NA | NA | NA | NA | NA |
| Rouette et al. 2020^77^ | Linked data | White | Lung | DPP-4i, GLP-1a, other antidiabetics | 30,598 | 63.47 | 4.60 | 59.44 | 5.46 | NA |
| Tseng et al. 2017^88^ | Claims data | Asian | Pancreas | DPP-4i, GLP-1a, other antidiabetics | 26,342 | 63.85 | NA | 50.50 | NA | NA |
| Tseng. 2016^83^ | Claims data | Asian | Pancreas | DPP-4i, other antidiabetics | 1,004,183 | 56.13 | NA | 52.96 | NA | NA |
| Tseng. 2017^85^ | Claims data | Asian | Breast | DPP-4i, other antidiabetics | 64,872 | 53.75 | NA | 0 | NA | NA |
| Tseng. 2017^86^ | Claims data | Asian | Prostate | DPP-4i, other antidiabetics | 75,584 | 51.55 | NA | NA | 6.15 | NA |
| Tseng. 2016^84^ | Claims data | Asian | Thyroid | DPP-4i, other antidiabetics | 115,318 | 56.95 | NA | 53.15 | 7 | NA |
| Tseng. 2017^87^ | Claims data | Asian | Oral Cavity | DPP-4i, other antidiabetics | 78,390 | 56 | NA | 54.35 | 6.60 | NA |
| Wang et al. 2022^91^ | EHR | White | Multiple cancer sites | GLP-1a, Metformin | 683,570 | NA | 5 | 50.17 | NA | NA |
| Williams et al. 2019^92^ | Linked data | White | Pancreas | DPP-4i, other antidiabetics | 757,804 | 64.23 | NA | 55.95 | 2.89 | NA |
| Wong et al. 2020^93^ | EHR | Asian | Multiple cancer sites | DPP-4i, TZDs, Insulin | 20,577 | 59.97 | 2.89 | 56.20 | 5.41 | 8.70 |
| Yang et al. 2024^94^ | Claims data | Asian | Liver | GLP-1a, Insulin | 14,342 | 49.20 | NA | 50.55 | NA | NA |
| **Case-Control studies** | | | | | | | | | | |
| Azoulay et al. 2016^11^ | EHR | White | Pancreas | DPP-4i, GLP-1a, SUs | 23,519 | 68.11 | 1.30 – 2.803 | 55 | 1.50 | NA |
| Chou et al. 2022^43^ | Claims data | Asian | Colorectal, Liver | DPP-4i, other antidiabetics | 9,690 | 63.4 | < 2 | 67.68 | NA | NA |
| Franchi et al. 2017^48^ | Linked data | White | Endometrial | DPP-4i, GLP-1a, other antidiabetics | 7,861 | 64 | 7.3 | NA | NA | NA |
| Garcia et al. 2021^53^ | Others | NA | Bladder | SGLT-2i, other antidiabetics | 198,258 | NA | NA | NA | NA | NA |
| Giorda et al. 2020^55^ | Claims data | White | Cholangiocarcinoma | DPP-4i, GLP-1a, other antidiabetics | 4,464 | 75.35 | NA | 49.60 | NA | NA |
| Lai et al. 2018^66^ | Claims data | Asian | Pancreas | DPP-4i, other antidiabetics | 2,444 | 67.85 | 4 | 54.80 | NA | NA |
| Shin et al. 2020^78^ | Claims data | Asian | Colorectal | DPP-4i, other antidiabetics | 8,456 | 61.35 | NA | 44.40 | NA | NA |
| Simo et al. 2013^80^ | EHR | White | Any type | DPP-4i, other antidiabetics | 3,056 | 72 | NA | 65.50 | 6.40 | 6.90 |
| Yang et al. 2022^95^ | Others | NA | Breast, Pancreas, Thyroid | GLP-1a, other antidiabetics | 601,491 | NA | NA | NA | NA | NA |

SGLT-2i: sodium-glucose cotransporter-2 inhibitors, DPP-4i: dipeptidyl peptidase-4 inhibitors, GLP-1a: glucagon-like peptide-1 agonists, TZDs: Thiazolidinediones, SUs: Sulfonylureas.^1^Only cohort studies were included in the NMA. ^2^NA: Not available in the study or unable to calculate due to missing data. ^3^Median or median range. ^4^pharmacovigilance databases. ^5^Electronic health records.

**Supplementary Table 4. Certainty of evidence (GRADE: Grading of Recommendations Assessment, Development and Evaluation).**

| **NMA Comparison** | | **Assessment of Direct estimate** | | | | **Assessment of Indirect estimate** | | **NMA estimates** | | | **Assessment of network estimate** | | |
| --- | --- | --- | --- | --- | --- | --- | --- | --- | --- | --- | --- | --- | --- |
| **Treatment 1** | **Treatment 2** | **Risk of bias^1^** | **Inconsistency** | **Indirectness** | **Publication bias^2^** | **Intransitivity** | **Imprecision** | **Point estimate** | **Lower limit** | **Upper limit** | **Incoherence** | **Imprecision** | **Final rating** |
| DPP-4i | Sulfonylureas | Not serious | Not serious | Not serious | Not serious | Not serious | Very serious | 0.76 | 0.6 | 0.96 | Not Serious | Not Serious | Low |
| Metformin | DPP-4i | Not serious | Not serious | Not serious | Not serious | Not serious | Very serious | 0.96 | 0.63 | 1.49 | Not Serious | Not Serious | Low |
| DPP-4i | GLP-1a | Not serious | Not serious | Not serious | Not serious | Not serious | Not Serious | 0.97 | 0.75 | 1.24 | Not Serious | Not Serious | Low |
| SGLT-2i | DPP-4i | Serious | Not serious | Not serious | Not serious | Not serious | Very serious | 0.72 | 0.57 | 0.92 | Not Serious | Not Serious | Very Low |
| DPP-4i | TZDs | Serious | Not serious | Not serious | Not serious | Not serious | Very serious | 0.95 | 0.68 | 1.35 | Not Serious | Not Serious | Very Low |
| Metformin | Sulfonylureas | Not serious | Not serious | Not serious | Not serious | Not serious | Very serious | 0.73 | 0.46 | 1.16 | Not Serious | Not Serious | Low |
| GLP-1a | Sulfonylureas | Not serious | Not serious | Not serious | Not serious | Not serious | Not Serious | 0.78 | 0.6 | 1.03 | Not Serious | Not Serious | Low |
| SGLT-2i | Sulfonylureas | Not serious | Not serious | Not serious | Not serious | Not serious | Not Serious | 0.54 | 0.40 | 0.74 | Not Serious | Not Serious | Low |
| TZDs | Sulfonylureas | Not serious | Not serious | Not serious | Not serious | Not serious | Not Serious | 0.80 | 0.54 | 1.18 | Not Serious | Not Serious | Very Low |
| Metformin | GLP-1a | Not serious | Not serious | Not serious | Not serious | Not serious | Very serious | 0.94 | 0.61 | 1.42 | Not Serious | Not Serious | Low |
| SGLT-2i | Metformin | Not serious | Not serious | Not serious | Not serious | Not serious | Not Serious | 0.75 | 0.47 | 1.17 | Not Serious | Not Serious | Very Low |
| Metformin | TZDs | Not serious | Not serious | Not serious | Not serious | Not serious | Very serious | 0.92 | 0.55 | 1.52 | Not Serious | Not Serious | Low |
| SGLT-2i | GLP-1a | Not serious | Not serious | Not serious | Not serious | Not serious | Not Serious | 0.70 | 0.53 | 0.92 | Not Serious | Not Serious | Low |
| GLP-1a | TZDs | Not serious | Not serious | Not serious | Not serious | Not serious | Not Serious | 0.98 | 0.68 | 1.43 | Not Serious | Not Serious | Low |
| SGLT-2i | TZDs | Serious | Not serious | Not serious | Not serious | Not serious | Not Serious | 0.68 | 0.46 | 1.02 | Not Serious | Not Serious | Very Low |

SGLT-2i: sodium-glucose cotransporter-2 inhibitors, DPP-4i: dipeptidyl peptidase-4 inhibitors, GLP-1a: glucagon-like peptide-1 agonists, TZDs: Thiazolidinediones.^1^The risk of bias of each direct estimate was (not serious) if most of the studies of this estimate had low or medium (ROBINS-I) bias risk. ^2^Egger test p-value < 0.05 for all direct comparisons with 3 studies or more.

**Supplementary Table 5. Quality assessment (Newcastle Ottawa Scale)**^1,2^.

| **Cohort Studies** | | | | | |
| --- | --- | --- | --- | --- | --- |
| **Study** | **Selection** | **Comparability** | **Outcome** | **Total Score** | **Assessment** |
| **Abrahami et al. 2018^37^** | **🟑🟑🟑🟑** | **🟑🟑** | **🟑🟑🟑** | 9 | High |
| **Abrahami et al. 2018^38^** | **🟑🟑🟑🟑** | **🟑🟑** | **🟑🟑🟑** | 9 | High |
| **Abrahami et al. 2022^13^** | **🟑🟑🟑🟑** | **🟑🟑** | **🟑🟑** | 8 | High |
| **Bea et al. 2024^39^** | **🟑🟑🟑🟑** | **🟑** | **🟑🟑🟑** | 8 | High |
| **Boniol et al. 2018^12^** | **🟑🟑🟑🟑** | **🟑** | **🟑🟑** | 7 | High |
| **Chan et al. 2023^40^** | **🟑🟑🟑🟑** | **🟑** | **🟑🟑🟑** | 8 | High |
| **Chen et al. 2023^41^** | **🟑🟑🟑🟑** | - | **🟑🟑🟑** | 7 | High |
| **Choi et al. 2019^42^** | **🟑🟑🟑🟑** | **🟑🟑** | **🟑🟑** | 8 | High |
| **Chou et al. 2023^44^** | **🟑🟑🟑🟑** | **🟑** | **🟑🟑** | 7 | High |
| **Chung et al. 2023^45^** | **🟑🟑🟑🟑** | **🟑** | **🟑🟑🟑** | 8 | High |
| **Dore et al. 2012^46^** | **🟑🟑🟑🟑** | **🟑** | **🟑🟑** | 7 | High |
| **Elashoff et al. 2011^47^** | **🟑** | - | - | 1 | Poor |
| **Funch et al. 2014^49^** | **🟑🟑🟑🟑** | - | **🟑🟑** | 6 | Medium |
| **Funch et al. 2018^50^** | **🟑🟑🟑🟑** | **🟑🟑** | **🟑🟑** | 8 | High |
| **Funch et al. 2019^52^** | **🟑🟑🟑🟑** | **🟑🟑** | **🟑🟑** | 8 | High |
| **Funch et al. 2021^51^** | **🟑🟑🟑🟑** | **🟑🟑** | **🟑🟑** | 8 | High |
| **Garry et al. 2018^54^** | **🟑🟑🟑🟑** | **🟑** | **🟑🟑** | 7 | High |
| **Gokhale et al. 2014^56^** | **🟑🟑🟑🟑** | **🟑** | **🟑🟑** | 7 | High |
| **Goossens et al. 2015^57^** | **🟑🟑🟑🟑** | **🟑** | **🟑🟑🟑** | 8 | High |
| **Hicks et al. 2016^58^** | **🟑🟑🟑🟑** | **🟑🟑** | **🟑🟑🟑** | 9 | High |
| **Hsu et al. 2021^59^** | **🟑🟑🟑🟑** | **🟑** | **🟑🟑** | 7 | High |
| **Htoo et al. 2016^60^** | **🟑🟑🟑🟑** | **🟑** | **🟑🟑** | 7 | High |
| **Hu et al. 2023^61^** | **🟑🟑🟑🟑** | **🟑** | **🟑🟑** | 7 | High |
| **Karp et al. 2019^62^** | **🟑🟑🟑🟑** | **🟑🟑** | **🟑🟑** | 8 | High |
| **Kim et al. 2023^63^** | **🟑🟑🟑🟑** | **🟑🟑** | **🟑🟑🟑** | 9 | High |
| **Knappen et al. 2016^64^** | **🟑🟑🟑🟑** | **🟑🟑** | **🟑🟑🟑** | 9 | High |
| **Kubota et al. 2023^65^** | **🟑🟑🟑🟑** | **🟑🟑** | **🟑🟑** | 8 | High |
| **Lee et al. 2018^67^** | **🟑🟑🟑🟑** | **🟑🟑** | **🟑🟑🟑** | 9 | High |
| **Lee et al. 2019^68^** | **🟑🟑🟑🟑** | **🟑🟑** | **🟑🟑🟑** | 9 | High |
| **Li et al. 2021^69^** | **🟑🟑🟑🟑** | **🟑** | **🟑🟑🟑** | 8 | High |
| **Liang et al. 2019^70^** | **🟑🟑🟑🟑** | **🟑🟑** | **🟑🟑** | 8 | High |
| **Lu et al. 2022^71^** | **🟑🟑🟑🟑** | **🟑🟑** | **🟑🟑🟑** | 9 | High |
| **Montvida et al. 2019^72^** | **🟑🟑🟑🟑** | **🟑** | **🟑🟑** | 7 | High |
| **Na et al. 2022^73^** | **🟑🟑🟑🟑** | **🟑🟑** | **🟑🟑🟑** | 9 | High |
| **Pradhan et al. 2023^74^** | **🟑🟑🟑🟑** | **🟑🟑** | **🟑🟑🟑** | 9 | High |
| **Rokszin et al. 2021^75^** | **🟑🟑🟑🟑** | **🟑** | **🟑🟑** | 7 | High |
| **Romley et al. 2012^76^** | **🟑🟑🟑🟑** | **🟑** | **🟑🟑** | 7 | High |
| **Rouette et al. 2020^77^** | **🟑🟑🟑🟑** | **🟑🟑** | **🟑🟑🟑** | 9 | High |
| **Shin et al. 2016^79^** | **🟑🟑🟑🟑** | **🟑** | **🟑🟑🟑** | 8 | High |
| **Suissa et al. 2021^81^** | **🟑🟑🟑🟑** | **🟑🟑** | **🟑🟑🟑** | 9 | High |
| **Suto et al. 2021^82^** | **🟑🟑🟑🟑** | **🟑** | **🟑🟑** | 7 | High |
| **Tseng. 2016^83^** | **🟑🟑🟑🟑** | **🟑** | **🟑🟑** | 7 | High |
| **Tseng. 2017^85^** | **🟑🟑🟑🟑** | **🟑** | **🟑🟑** | 7 | High |
| **Tseng. 2016^84^** | **🟑🟑🟑🟑** | **🟑** | **🟑🟑** | 7 | High |
| **Tseng. 2017^86^** | **🟑🟑🟑🟑** | **🟑** | **🟑🟑** | 7 | High |
| **Tseng. 2017^87^** | **🟑🟑🟑🟑** | **🟑🟑** | **🟑🟑** | 8 | High |
| **Tseng et al. 2017^88^** | **🟑🟑🟑🟑** | **🟑** | **🟑🟑** | 7 | High |
| **Ueda et al. 2021^90^** | **🟑🟑🟑🟑** | **🟑🟑** | **🟑🟑** | 8 | High |
| **Ueda et al. 2022^89^** | **🟑🟑🟑🟑** | **🟑🟑** | **🟑🟑** | 8 | High |
| **Wang et al. 2022^91^** | **🟑🟑🟑🟑** | **🟑🟑** | **🟑🟑🟑** | 9 | High |
| **Williams et al. 2019^92^** | **🟑🟑🟑🟑** | **🟑🟑** | **🟑🟑** | 8 | High |
| **Wong et al. 2020^93^** | **🟑🟑🟑🟑** | **🟑** | **🟑🟑** | 7 | High |
| **Yang et al. 2024^94^** | **🟑🟑🟑🟑** | **🟑🟑** | **🟑🟑🟑** | 9 | High |
| **Case-Control studies** | | | | | |
|  | **Selection** | **Comparability** | **Exposure** | **Total Score** | **Assessment** |
| **Azoulay et al. 2016^11^** | **🟑🟑🟑** | **🟑🟑** | **🟑🟑🟑** | 8 | High |
| **Chou et al. 2022^43^** | **🟑🟑🟑** | **🟑** | **🟑🟑🟑** | 7 | High |
| **Franchi et al. 2017^48^** | **🟑🟑🟑** | **🟑** | **🟑🟑🟑** | 7 | High |
| **Garcia et al. 2021^53^** | **🟑🟑** | - | **🟑** | 3 | Poor |
| **Giorda et al. 2020^55^** | **🟑🟑🟑** | **🟑** | **🟑🟑🟑** | 7 | High |
| **Lai et al. 2018^66^** | **🟑🟑🟑** | **🟑** | **🟑🟑🟑** | 7 | High |
| **Shin et al. 2020^78^** | **🟑🟑🟑** | **🟑🟑** | **🟑🟑🟑** | 8 | High |
| **Simo et al. 2013^80^** | **🟑🟑🟑** | **🟑🟑** | **🟑🟑🟑** | 8 | High |
| **Yang et al. 2022^95^** | **🟑🟑** | - | **🟑** | 3 | Poor |

^1^For the comparability section, the studies were assigned one point if they controlled for the major cancer risk factors. One extra point was given to studies that controlled for additional factors like duration of diabetes, HbA1c, and other medications. ^2^The studies were rated on a scale from 0 to 9, with ratings of 0 to 3 considered as poor quality, 4 to 6 as medium quality, and 7 to 9 as high quality.

**Supplementary Table 6. Risk of bias (ROBINS-I: Risk Of Bias In Non-randomised Studies - of Interventions) ^1,2^.**

| **Cohort Studies** | | | | | | | | |
| --- | --- | --- | --- | --- | --- | --- | --- | --- |
| **Study** | **Domain 1** | **Domain 2** | **Domain 3** | **Domain 4** | **Domain 5** | **Domain 6** | **Domain 7** | **Overall Assessment** |
| **Abrahami et al. 2018^37^** | Low | Medium | Low | Low | Low | Low | Low | Medium |
| **Abrahami et al. 2018^38^** | Low | Medium | Low | Low | Low | Low | Low | Medium |
| **Abrahami et al. 2022^13^** | Low | Medium | Low | Low | Low | Low | Low | Medium |
| **Bea et al. 2024^39^** | Serious | Low | Low | Low | Low | Low | Low | Serious |
| **Boniol et al. 2018^12^** | Serious | Low | Low | Low | Low | Low | Low | Serious |
| **Chan et al. 2023^40^** | Serious | Low | Low | Low | Serious | Low | Low | Serious |
| **Chen et al. 2023^41^** | Serious | Low | Low | Low | Low | Low | Low | Serious |
| **Choi et al. 2019^42^** | Low | Medium | Low | Low | Low | Low | Low | Medium |
| **Chou et al. 2023^44^** | Serious | Low | Low | Low | Low | Low | Low | Serious |
| **Chung et al. 2023^45^** | Serious | Low | Low | Low | Medium | Low | Low | Serious |
| **Dore et al. 2012^46^** | Serious | Low | Low | Low | Low | Low | Low | Serious |
| **Elashoff et al. 2011^47^** | Critical | Critical | Low | Low | Low | Critical | Low | Critical |
| **Funch et al. 2014^49^** | Critical | Medium | Low | Low | Low | Low | Low | Critical |
| **Funch et al. 2018^50^** | Low | Low | Low | Low | Low | Low | Low | Low |
| **Funch et al. 2019^52^** | Low | Low | Low | Low | Low | Low | Low | Low |
| **Funch et al. 2021^51^** | Low | Low | Low | Low | Low | Low | Low | Low |
| **Garry et al. 2018^54^** | Serious | Low | Low | Low | Low | Low | Low | Serious |
| **Gokhale et al. 2014^56^** | Serious | Medium | Low | Low | Low | Low | Low | Serious |
| **Goossens et al. 2015^57^** | Serious | Medium | Low | Low | Low | Low | Low | Serious |
| **Hicks et al. 2016^58^** | Low | Medium | Low | Low | Low | Low | Low | Medium |
| **Hsu et al. 2021^59^** | Serious | Low | Low | Low | Low | Low | Low | Serious |
| **Htoo et al. 2016^60^** | Serious | Medium | Low | Low | Low | Low | Low | Serious |
| **Hu et al. 2023^61^** | Serious | Low | Low | Low | Low | Low | Low | Serious |
| **Karp et al. 2019^62^** | Low | Medium | Low | Low | Low | Low | Low | Medium |
| **Kim et al. 2023^63^** | Low | Low | Low | Low | Low | Low | Low | Low |
| **Knappen et al. 2016^64^** | Low | Medium | Low | Low | Low | Low | Low | Medium |
| **Kubota et al. 2023^65^** | Low | Low | Low | Low | Low | Low | Low | Low |
| **Lee et al. 2018^67^** | Low | Low | Low | Low | Low | Low | Low | Low |
| **Lee et al. 2019^68^** | Low | Low | Low | Low | Low | Low | Low | Low |
| **Li et al. 2021^69^** | Serious | Low | Low | Low | Low | Low | Low | Serious |
| **Liang et al. 2019^70^** | Low | Low | Low | Low | Low | Low | Low | Low |
| **Lu et al. 2022^71^** | Low | Medium | Low | Low | Low | Low | Low | Medium |
| **Montvida et al. 2019^72^** | Serious | Medium | Low | Low | Low | Low | Low | Serious |
| **Na et al. 2022^73^** | Low | Low | Low | Low | Low | Low | Low | Low |
| **Pradhan et al. 2023^74^** | Low | Low | Low | Low | Low | Low | Low | Low |
| **Rokszin et al. 2021^75^** | Serious | Low | Low | Low | Low | Low | Low | Serious |
| **Romley et al. 2012^76^** | Serious | Low | Low | Low | Low | Low | Low | Serious |
| **Rouette et al. 2020^77^** | Low | Medium | Low | Low | Low | Low | Low | Medium |
| **Shin et al. 2016^79^** | Serious | Low | Low | Low | Low | Low | Low | Serious |
| **Suissa et al. 2021^81^** | Low | Low | Low | Low | Low | Low | Low | Low |
| **Suto et al. 2021^82^** | Serious | Low | Low | Low | Low | Low | Low | Serious |
| **Tseng. 2016^83^** | Serious | Low | Low | Low | Low | Low | Low | Serious |
| **Tseng. 2017^85^** | Serious | Low | Low | Low | Low | Low | Low | Serious |
| **Tseng. 2016^84^** | Serious | Low | Low | Low | Low | Low | Low | Serious |
| **Tseng. 2017^86^** | Serious | Low | Low | Low | Low | Low | Low | Serious |
| **Tseng. 2017^87^** | Low | Low | Low | Low | Low | Low | Low | Low |
| **Tseng et al. 2017^88^** | Serious | Low | Low | Low | Low | Low | Low | Serious |
| **Ueda et al. 2021^90^** | Low | Medium | Low | Low | Low | Low | Low | Medium |
| **Ueda et al. 2022^89^** | Low | Medium | Low | Low | Low | Low | Low | Medium |
| **Wang et al. 2022^91^** | Low | Medium | Low | Low | Low | Low | Low | Medium |
| **Williams et al. 2019^92^** | Low | Medium | Low | Low | Low | Low | Low | Medium |
| **Wong et al. 2020^93^** | Serious | Medium | Low | Low | Low | Low | Low | Serious |
| **Yang et al. 2024^94^** | Low | Low | Low | Low | Low | Low | Low | Low |
| **Case-Control studies** | | | | | | | | |
|  | **Domain 1** | **Domain 2** | **Domain 3** | **Domain 4** | **Domain 5** | **Domain 6** | **Domain 7** | **Overall Assessment** |
| **Azoulay et al. 2016^11^** | Low | Low | Low | Low | Low | Low | Low | Low |
| **Chou et al. 2022^43^** | Serious | Low | Low | Low | Low | Low | Low | Serious |
| **Franchi et al. 2017^48^** | Serious | Low | Low | Low | Low | Low | Low | Serious |
| **Garcia et al. 2021^53^** | Critical | Critical | Low | Low | Low | Critical | Low | Critical |
| **Giorda et al. 2020^55^** | Serious | Low | Low | Low | Low | Low | Low | Serious |
| **Lai et al. 2018^66^** | Low | Low | Low | Low | Low | Low | Low | Low |
| **Shin et al. 2020^78^** | Low | Low | Low | Low | Low | Low | Low | Low |
| **Simo et al. 2013^80^** | Low | Low | Low | Low | Low | Low | Low | Low |
| **Yang et al. 2022^95^** | Critical | Critical | Low | Low | Low | Critical | Low | Critical |

^1^Grades are (Low / Moderate / Serious / Critical). The seven domains of bias are: Domain 1: Confounding, Domain 2: Selection bias, Domain 3: Bias in measurement classification of interventions, Domain 4: Bias due to deviations from intended interventions, Domain 5: Bias due to missing data, Domain 6: Bias in measurement of outcomes, Domain 7: Bias in selection of the reported result. ^2^A low risk of confounding was considered if the study controlled for the major cancer risk factors, including age, gender, previous cancers, smoking, alcohol use, BMI (or appropriate proxies), in addition to any additional diabetes related covariates that made the study comparable to a well-performed RCT. A low risk of selection bias was considered if the two groups had relatively equal parameters like follow-up times, male percentage, HbA1c, and duration of diabetes. The study had an overall low risk of bias if it had a low-risk level in all seven domains.

**Supplementary Table 7. The number of cancer events within each comparison (overall risk of cancer).**

| **Num.** | **Treatment Name** | **DPP-4i** | | **TZDs** | | **GLP-1a** | | **SGLT-2i** | | **Sulfonylureas** | | **Metformin** | |
| --- | --- | --- | --- | --- | --- | --- | --- | --- | --- | --- | --- | --- | --- |
|  | **Study Name** | **Events** | **Patients** | **Events** | **Patients** | **Events** | **Patients** | **Events** | **Patients** | **Events** | **Patients** | **Events** | **Patients** |
| **1** | **Abrahami et al. 2022 (Bladder)^14^** |  |  |  |  | 932 | 375997 | 1046 | 453560 |  |  |  |  |
|  | **Abrahami et al. 2022 (Bladder)** | 3421 | 853186 |  |  |  |  | 743 | 347059 |  |  |  |  |
| **2** | **Abrahami et al. 2018 (colorectal)^38^** | 117 | 6002 |  |  |  |  |  |  | 241 | 18513 |  |  |
|  | **Abrahami et al. 2018 (colorectal)** |  |  |  |  | 26 | 1177 |  |  | 302 | 18513 |  |  |
| **3** | **Bea et al. 2024 (Thyroid)^39^** | 1386 | 904300 |  |  |  |  | 123 | 112017 |  |  |  |  |
|  | **Bea et al. 2024 (Thyroid)** |  |  |  |  | 23 | 21722 | 524 | 326993 |  |  |  |  |
| **4** | **Chan et al. 2023 (colorectal)^40^** | 106 | 12659 |  |  |  |  | 66 | 12659 |  |  |  |  |
| **5** | **Choi et al. 2019 (All)^42^** | 20 | 769 |  |  |  |  |  |  |  |  | 33 | 769 |
| **6** | **Chou et al. 2023 (Pancreas)^44^** | 42 | 6479 |  |  |  |  | 10 | 6479 |  |  |  |  |
| **7** | **Chung et al. 2022 (All)^45^** | 432 | 18167 |  |  |  |  | 242 | 18167 |  |  |  |  |
| **8** | **Funch et al. 2018 (Breast)^50^** | 62 | 12173 |  |  | 67 | 12173 |  |  |  |  |  |  |
|  | **Funch et al. 2018 (Breast)** |  |  |  |  | 56 | 11934 |  |  |  |  | 52 | 11934 |
|  | **Funch et al. 2018 (Breast)** |  |  |  |  | 53 | 12418 |  |  | 54 | 12418 |  |  |
|  | **Funch et al. 2018 (Breast)** |  |  | 41 | 8009 | 53 | 8009 |  |  |  |  |  |  |
| **9** | **Funch et al. 2021 (Thyroid)^51^** | 16 | 25579 |  |  | 24 | 25579 |  |  |  |  |  |  |
|  | **Funch et al. 2021 (Thyroid)** |  |  |  |  | 23 | 24747 |  |  |  |  | 23 | 24747 |
|  | **Funch et al. 2021 (Thyroid)** |  |  |  |  | 23 | 26076 |  |  | 17 | 26076 |  |  |
|  | **Funch et al. 2021 (Thyroid)** |  |  | 17 | 18210 | 18 | 18210 |  |  |  |  |  |  |
| **10** | **Garry et al. 2018 (Bladder)^54^** | 193 | 61438 | 147 | 29651 |  |  |  |  |  |  |  |  |
| **11** | **Gokhale et al. 2014 (Pancreas)^56^** | 26 | 18179 |  |  |  |  |  |  | 177 | 63746 |  |  |
|  | **Gokhale et al. 2014 (Pancreas)** | 52 | 29366 | 54 | 26332 |  |  |  |  |  |  |  |  |
| **12** | **Hicks et al. 2016 (Breast)^58^** | 68 | 2422 |  |  | 31 | 498 |  |  |  |  |  |  |
| **13** | **Htoo et al. 2016 (colorectal)^60^** | 104 | 39334 | 63 | 25786 |  |  |  |  |  |  |  |  |
|  | **Htoo et al. 2016 (colorectal)** | 73 | 27047 |  |  |  |  |  |  | 266 | 76012 |  |  |
| **14** | **Kubota et al. 2023 (Pancreas)^65^** | 142 | 61430 | 6 | 3329 |  |  | 11 | 9221 | 13 | 4774 | 22 | 17324 |
| **15** | **Li et al. 2021 (Bladder)^69^** |  |  | 4 | 10547 |  |  | 1 | 3359 |  |  |  |  |
| **16** | **Lu et al. 2022 (Prostate)^71^** |  |  |  |  | 34 | 5063 |  |  | 2157 | 112955 |  |  |
|  | **Lu et al. 2022 (Prostate)** | 611 | 53529 |  |  |  |  |  |  | 2208 | 114417 |  |  |
| **17** | **Pradhan et al. 2023 (Melanoma)^74^** | 119 | 96739 |  |  |  |  |  |  | 515 | 209341 |  |  |
|  | **Pradhan et al. 2023 (non-Melanoma)** | 1059 | 96411 |  |  |  |  |  |  | 3643 | 208626 |  |  |
| **18** | **Shin et al. 2016 (Pancreas)^79^** | 2 | 1620 |  |  |  |  |  |  |  |  | 4 | 3240 |
| **19** | **Suissa et al. 2021 (Breast)^81^** | 382 | 36631 |  |  |  |  | 67 | 9938 |  |  |  |  |
| **20** | **Suto et al. 2021 (All)^82^** | 506 | 18316 |  |  |  |  | 408 | 18383 |  |  |  |  |
| **21** | **Ueda et al. 2022 (Bladder)^89^** |  |  |  |  | 70 | 49398 | 73 | 57383 |  |  |  |  |
|  | **Ueda et al. 2022 (Renal)** |  |  |  |  | 58 | 49404 | 64 | 57393 |  |  |  |  |
| **22** | **Ueda et al. 2021 (Cholangiocarcinoma)^90^** | 222 | 222577 |  |  |  |  |  |  | 128 | 123908 |  |  |
|  | **Ueda et al. 2021 (Cholangiocarcinoma)** |  |  |  |  | 92 | 96813 |  |  | 157 | 142578 |  |  |

SGLT-2i: sodium-glucose cotransporter-2 inhibitors, DPP-4i: dipeptidyl peptidase-4 inhibitors, GLP-1a: glucagon-like peptide-1 agonists, TZDs: Thiazolidinediones.

**Supplementary Table 8. The number of site-specific cancer events within each comparison.**

| **Gastrointestinal Cancer** | | | | | | | | | | | | | |
| --- | --- | --- | --- | --- | --- | --- | --- | --- | --- | --- | --- | --- | --- |
| **Number** | **Treatment Name** | **DPP-4i** | | **TZDs** | | **GLP-1a** | | **SGLT-2i** | | **Sulfonylureas** | | **Metformin** | |
|  | **Study Name** | **Events** | **Patients** | **Events** | **Patients** | **Events** | **Patients** | **Events** | **Patients** | **Events** | **Patients** | **Events** | **Patients** |
| **1** | **Abrahami et al. 2018 (Colorectal)^38^** | 117 | 6002 |  |  |  |  |  |  | 241 | 18513 |  |  |
|  | **Abrahami et al. 2018 (Colorectal)** |  |  |  |  | 26 | 1177 |  |  | 302 | 18513 |  |  |
| **2** | **Chan et al. 2023 (Colorectal)^40^** | 106 | 12659 |  |  |  |  | 66 | 12659 |  |  |  |  |
| **3** | **Choi et al. 2019 (Gastrointestinal)^42^** | 9 | 769 |  |  |  |  |  |  |  |  | 17 | 769 |
| **4** | **Chou et al. 2023 (Pancreas)^44^** | 42 | 6479 |  |  |  |  | 10 | 6479 |  |  |  |  |
| **5** | **Chung et al. 2022 (Gastrointestinal)^45^** | 196 | 18167 |  |  |  |  | 129 | 18167 |  |  |  |  |
| **6** | **Gokhale et al. 2014 (Pancreas)^56^** | 26 | 18179 |  |  |  |  |  |  | 177 | 63746 |  |  |
|  | **Gokhale et al. 2014 (Pancreas)** | 52 | 29366 | 54 | 26332 |  |  |  |  |  |  |  |  |
| **7** | **Htoo et al. 2016 (Colorectal)^60^** | 104 | 39334 | 63 | 25786 |  |  |  |  |  |  |  |  |
|  | **Htoo et al. 2016 (Colorectal)** | 73 | 27047 |  |  |  |  |  |  | 266 | 76012 |  |  |
| **8** | **Kubota et al. 2023 (Pancreas)^65^** | 142 | 61430 | 6 | 3329 |  |  | 11 | 9221 | 13 | 4774 | 22 | 17324 |
| **9** | **Rokszin et al. 2021 (Lower GI)^75^** | 59 | 17869 |  |  |  |  | 48 | 18023 |  |  |  |  |
|  | **Rokszin et al. 2021 (Rectum)** | 23 | 17833 |  |  |  |  | 25 | 18000 |  |  |  |  |
|  | **Rokszin et al. 2021 (Pancreas)** | 30 | 17840 |  |  |  |  | 34 | 18009 |  |  |  |  |
| **10** | **Shin et al. 2016 (Pancreas)^79^** | 2 | 1620 |  |  |  |  |  |  |  |  | 4 | 3240 |
| **11** | **Ueda et al. 2021 (Cholangiocarcinoma)^90^** | 222 | 222577 |  |  |  |  |  |  | 128 | 123908 |  |  |
|  | **Ueda et al. 2021 (Cholangiocarcinoma)** |  |  |  |  | 92 | 96813 |  |  | 157 | 142578 |  |  |
| **Breast Cancer** | | | | | | | | | | | | | |
| **Number** | **Treatment Name** | **DPP-4i** | | **TZDs** | | **GLP-1a** | | **SGLT-2i** | | **Sulfonylureas** | | **Metformin** | |
|  | **Study Name** | **Events** | **Patients** | **Events** | **Patients** | **Events** | **Patients** | **Events** | **Patients** | **Events** | **Patients** | **Events** | **Patients** |
| **1** | **Choi et al. 2019^42^** | 3 | 333 |  |  |  |  |  |  |  |  | 3 | 330 |
| **2** | **Chung et al. 2022^45^** | 69 | 18167 |  |  |  |  | 32 | 18167 |  |  |  |  |
| **3** | **Funch et al. 2018^50^** | 62 | 12173 |  |  | 67 | 12173 |  |  |  |  |  |  |
|  | **Funch et al. 2018** |  |  |  |  | 56 | 11934 |  |  |  |  | 52 | 11934 |
|  | **Funch et al. 2018** |  |  |  |  | 53 | 12418 |  |  | 54 | 12418 |  |  |
|  | **Funch et al. 2018** |  |  | 41 | 8009 | 53 | 8009 |  |  |  |  |  |  |
| **4** | **Hicks et al. 2016^58^** | 68 | 2422 |  |  | 31 | 498 |  |  |  |  |  |  |
| **5** | **Rokszin et al. 2021^75^** | 50 | 17860 |  |  |  |  | 43 | 18018 |  |  |  |  |
| **6** | **Suissa et al. 2021^81^** | 382 | 36631 |  |  |  |  | 67 | 9938 |  |  |  |  |
| **Head/neck and respiratory/thoracic Cancer** | | | | | | | | | | | | | |
| **Number** | **Treatment Name** | **DPP-4i** | | **TZDs** | | **GLP-1a** | | **SGLT-2i** | | **Sulfonylureas** | | **Metformin** | |
|  | **Study Name** | **Events** | **Patients** | **Events** | **Patients** | **Events** | **Patients** | **Events** | **Patients** | **Events** | **Patients** | **Events** | **Patients** |
| **1** | **Bea et al. 2024 (Thyroid)^39^** | 1386 | 904300 |  |  |  |  | 123 | 112017 |  |  |  |  |
|  | **Bea et al. 2024 (Thyroid)** |  |  |  |  | 23 | 21722 | 524 | 326993 |  |  |  |  |
| **2** | **Choi et al. 2019 (Thymus and Lung)^42^** | 1 | 769 |  |  |  |  |  |  |  |  | 6 | 769 |
| **3** | **Chung et al. 2022 (Lung)^45^** | 82 | 18167 |  |  |  |  | 42 | 18167 |  |  |  |  |
| **4** | **Funch et al. 2021 (Thyroid)^51^** | 16 | 25579 |  |  | 24 | 25579 |  |  |  |  |  |  |
|  | **Funch et al. 2021 (Thyroid)** |  |  |  |  | 23 | 24747 |  |  |  |  | 23 | 24747 |
|  | **Funch et al. 2021 (Thyroid)** |  |  |  |  | 23 | 26076 |  |  | 17 | 26076 |  |  |
|  | **Funch et al. 2021 (Thyroid)** |  |  | 17 | 18210 | 18 | 18210 |  |  |  |  |  |  |
| **5** | **Rokszin et al. 2021 (Lung and Larynx)^75^** | 61 | 17871 |  |  |  |  | 53 | 18028 |  |  |  |  |
| **Genitourinary Cancer** | | | | | | | | | | | | | |
| **Number** | **Treatment Name** | **DPP-4i** | | **TZDs** | | **GLP-1a** | | **SGLT-2i** | | **Sulfonylureas** | |  |  |
|  | **Study Name** | **Events** | **Patients** | **Events** | **Patients** | **Events** | **Patients** | **Events** | **Patients** | **Events** | **Patients** |  |  |
| **1** | **Abrahami et al. 2022 (Bladder)^13^** |  |  |  |  | 932 | 375997 | 1046 | 453560 |  |  |  |  |
|  | **Abrahami et al. 2022 (Bladder)** | 3421 | 853186 |  |  |  |  | 743 | 347059 |  |  |  |  |
| **2** | **Chung et al. 2022 (Bladder)^45^** | 39 | 18167 |  |  |  |  | 11 | 18167 |  |  |  |  |
|  | **Chung et al. 2022 (Genitourinary)** | 82 | 18167 |  |  |  |  | 39 | 18167 |  |  |  |  |
| **3** | **Garry et al. 2018 (Bladder)^54^** | 193 | 61438 | 147 | 29651 |  |  |  |  |  |  |  |  |
| **4** | **Li et al. 2021 (Bladder)^69^** |  |  | 4 | 10547 |  |  | 1 | 3359 |  |  |  |  |
| **5** | **Lu et al. 2022 (Prostate)^71^** |  |  |  |  | 34 | 5063 |  |  | 2157 | 112955 |  |  |
|  | **Lu et al. 2022 (Prostate)** | 611 | 53529 |  |  |  |  |  |  | 2208 | 114417 |  |  |
| **6** | **Rokszin et al. 2021 (Prostate)^75^** | 46 | 17856 |  |  |  |  | 33 | 18008 |  |  |  |  |
|  | **Rokszin et al. 2021 (Urinary tract)** | 54 | 17864 |  |  |  |  | 29 | 18004 |  |  |  |  |
| **7** | **Ueda et al. 2022 (Bladder)^89^** |  |  |  |  | 70 | 49398 | 73 | 57383 |  |  |  |  |
|  | **Ueda et al. 2022 (Kidney)** |  |  |  |  | 58 | 49404 | 64 | 57393 |  |  |  |  |

SGLT-2i: sodium-glucose cotransporter-2 inhibitors, DPP-4i: dipeptidyl peptidase-4 inhibitors, GLP-1a: glucagon-like peptide-1 agonists, TZDs: Thiazolidinediones.

**Supplementary Figure 1. League table of the overall risk of cancer for all pairwise class comparisons.**

| **SGLT-2i** |  |  |  |  |  |
| --- | --- | --- | --- | --- | --- |
| 0.75 (0.47 – 1.17) | **Metformin** |  |  |  |  |
| 0.72 (0.57 – 0.90) | 0.96 (0.63 – 1.47) | **DPP-4i** |  |  |  |
| 0.70 (0.53 – 0.91) | 0.93 (0.61 – 1.42) | 0.97 (0.76 – 1.24) | **GLP-1a** |  |  |
| 0.67 (0.46 – 1.00) | 0.90 (0.54 – 1.52) | 0.94 (0.67 – 1.33) | 0.97 (0.67 – 1.42) | **TZDs** |  |
| 0.54 (0.40 – 0.73) | 0.72 (0.46 – 1.13) | 0.75 (0.60 – 0.95) | 0.77 (0.60 – 1.01) | 0.80 (0.54 – 1.18) | **Sulfonylureas** |

SGLT-2i: sodium-glucose cotransporter-2 inhibitors, DPP-4i: dipeptidyl peptidase-4 inhibitors, GLP-1a: glucagon-like peptide-1 agonists, TZDs: Thiazolidinediones.

**Supplementary Figure 2. Rankogram and SUCRA scores for the overall risk of cancer NMA.**

SGLT-2i: sodium-glucose cotransporter-2 inhibitors, DPP-4i: dipeptidyl peptidase-4 inhibitors, GLP-1a: glucagon-like peptide-1 agonists, TZDs: Thiazolidinediones.

**Supplementary Figure 3. Inconsistency graph for the overall risk of cancer NMA.**

**Supplementary Figure 4. Forest plot for risk of genitourinary cancer NMA.**

SGLT-2i: sodium-glucose cotransporter-2 inhibitors, DPP-4i: dipeptidyl peptidase-4 inhibitors, GLP-1a: glucagon-like peptide-1 agonists, TZDs: Thiazolidinediones.

**Supplementary Figure 5. Forest plot for risk of breast cancer NMA.**

SGLT-2i: sodium-glucose cotransporter-2 inhibitors, DPP-4i: dipeptidyl peptidase-4 inhibitors, GLP-1a: glucagon-like peptide-1 agonists, TZDs: Thiazolidinediones.
